# Sweat the small stuff: A review of the use of accelerometers to estimate energy expenditure in wild animals

**DOI:** 10.1111/1365-2656.70162

**Published:** 2025-11-08

**Authors:** Kyle H. Elliott

**Affiliations:** ^1^ Department of Natural Resource Sciences McGill University Montreal Quebec Canada

**Keywords:** accelerometer, accelerometry, animal energetics, biologging, daily energy expenditure, doubly labelled water, heart rate, telemetry

## Abstract

1. Dynamic body acceleration (DBA), a measure of work based on Newtonian biomechanics, is a metric often used to estimate daily energy expenditure (DEE) with accelerometers, but studies validating DBA in wild animals have shown mixed results.
2. I review all studies using accelerometry to measure energy expenditure in free‐living (wild or farmed) animals, focussing on those that calibrate DBA against doubly labelled water or heart rate as the ‘gold standard’ for DEE.
3. Most (~90%) energetics studies using DBA focus on endotherms, even though DBA may work better for ectotherms. In nearly all studies of endotherms, average DBA increased linearly with DEE, but the intercept of the DBA‐DEE relationship was not constant across contexts—even within the same species. DBA‐DEE relationships were stronger with mass‐specific DEE and slightly stronger with vectorial DBA than overall DBA.
4. In a case study of six seabird species with similar activity modes and physiologies, DBA‐DEE slopes varied with activity, but were consistent across species for flight, implying that activity‐specific slopes might be applied across species in some cases where sufficient sampling occurs within similar taxa.
5. I offer recommendations for the use of DBA to estimate DEE. I explore the potential for a general physiological model across species, without species‐specific calibrations, and note where the DBA‐DEE relationship breaks down and where we need more data. DBA, used under appropriate conditions, is an index of energy use in many endotherms, and I encourage its use with more ecological questions.

## Graphical abstract

The growing use of accelerometry by ecologists to measure energy expenditure can make physiologists sweat due to its many pitfalls. This review comprehensively describes the use of accelerometry in wild animals, the successes (and failures) of calibrations and how best to estimate energy expenditure from accelerometers. The review will accelerate the adoption of best practices so the field can continue to gain momentum.

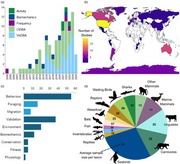

## 1 INTRODUCTION

‘The change of motion is proportional to the motive force impressed and takes place along the straight line in which that force is impressed’.—Isaac Newton, *Principia Mathematica*

Energy is the fundamental currency of life, and the balance between energy expended and gained determines animal fitness (Grémillet et al., 2018; Speakman, 1997). However, ecologists have limited tools to estimate energy expenditure in wild animals. Indeed, although the current ‘golden age of biologging’ has revealed the behaviour and location of millions of animals, we often cannot measure the energy costs that are critical for determining why they do what they do or why they are where they are (Wilmers et al., 2015). This problem is being resolved as miniature accelerometers are now integrated into many biologgers, and accelerometry is a widely used technique for estimating energy costs (Wilson et al., 2020).

Methods that estimate the energy expenditure of wild animals using biologgers include heart rate telemetry, energy‐time budget modelling and accelerometry (Green, 2011; Wilmers et al., 2015). Doubly labelled water (DLW), heart rate and respirometry are used to calibrate those methods (Green, 2011; Shaffer, 2011; Wilson et al., 2020). DLW requires recapturing the animal or at least its tissues (i.e. faeces) and only provides an average value over the scale of days, while respirometry can only be used in semi‐captive situations (i.e. burrows, individuals trained to breathe into masks), and so those techniques are limited in their application in wild animals (Halsey, 2017; Speakman, 1997). Heart rate telemetry is the most‐validated method for biologging of wild animal energy expenditure but is logistically challenging due to the placement of external electrodes or surgical implantation and relies on assumptions about cardiac output that are difficult to validate (Green, 2011). Modelling approaches typically rely on using biologgers to develop activity budgets and then apply activity‐specific metabolic rates to those activities, yet activities seldom have a single metabolic rate; the average metabolic rate for walking, for example, will change depending on substrate (Speakman, 1997; Wilson et al., 2020). Other techniques, such as thermal imaging and counting breaths, can only be applied in limited situations (Tattersall, 2016; Wilson, 2003). Given the above constraints on other methods, accelerometry has become a widespread technique for estimating energy expenditure by biologging but has had few validations in the wild and is subject to several caveats (Wilson et al., 2020).

Accelerometry is a method for estimating energy expenditure in wild animals that typically uses dynamic body acceleration (DBA), based on Newton's Second Law, as described in the quote at the start of this paper (Gleiss et al., 2011; Wilson et al., 2006). The accelerometer records total acceleration across three axes, which includes both the dynamic component from an animal's movement and the static component from gravity (posture). DBA is the area under the accelerometer–time curve after subtracting the static component (Wilson et al., 2006). Specifically, DBA is related to work (*W*), which is equal to the integral of Force (*F*) over distance (*x*):

(1) W=∫Fdx=mv∫adt=mvDBA

and therefore mass‐specific energy expended at a constant speed (*v*) is proportional to DBA, provided all work is in the direction of travel (Gleiss et al., 2011; Stothart et al., 2016). If work is measured over a defined time, this becomes an estimate of power (e.g. Equation 7 in Gleiss et al., 2011). DBA is calculated by summing across all axes the absolute values of DBA (overall DBA, ODBA) or the square root of the sum of the squares of DBA (vectorial DBA, VeDBA) (Qasem et al., 2012).

Accelerometers are widely used to estimate energy expenditure in humans, and most of us are now familiar with smartwatch or smartphone apps that estimate our caloric output. Algorithms use the accelerometer data from smartphones to create time‐activity budgets (time spent in each activity; Lyden et al., 2011) based on past calibrations where the metabolic rate of individuals wearing those devices was measured using respirometry or DLW, developing calibration coefficients called Metabolic Equivalent of Tasks (METs). For example, time budgets may be divided into sedentary (e.g. sitting, 1 MET), moderate (e.g. walking, 2–3 METs) and vigorous (e.g. running, 6+ METs) activities (Kozey et al., 2010; Lyden et al., 2011). METs are either applied via cut‐point models where an average MET is applied to each task based on the average count of g's in the activity or via regression equations where counts of g's within tasks are used to estimate costs within tasks (Kozey et al., 2010; Lyden et al., 2011). The counting of g's is analogous to summing g over time to calculate DBA, and the METs represent the slope of the DBA–metabolic rate relationship. The METs are then applied within the smartphone app to determine the caloric output of any individual carrying the smartphone, sometimes after including individual data (age, mass, sex, height). The same idea applies to wild animals (Wilson et al., 2006, 2020).

Determining METs for each activity in wild animals is more challenging as there are many activities that cannot be directly calibrated against respirometry or DLW, which is, of course, one reason why DBA is often used as a proxy for energy expenditure (Wilson et al., 2006). The first application of accelerometry to estimate energy expenditure occurred in Weddell seals (*Leptonychotes weddellii*) diving in isolated holes in Antarctic ice, where the number of flipper strokes correlated with oxygen consumed in the subsequent surface period (Williams et al., 2004). Next, the DBA approach was developed in a study where ODBA correlated with oxygen consumption rate based on cormorants walking on a treadmill (Wilson et al., 2006). Since then, ODBA has been validated as a proxy for oxygen consumption across many different captive animals (Elliott, 2016; Green et al., 2009; Halsey et al., 2009, 2011). Wild animals experience more variation in environment and activity type than animals on treadmills, yet many studies have now used heart rate or DLW as ‘gold standards’ to show that DBA is indeed a proxy of daily energy expenditure (DEE) in the wild (Duriez et al., 2014; Elliott et al., 2013; Hicks et al., 2017).

Some validations have inflated the link between DBA and energy expenditure by falling into Halsey's Time Trap, where DBA and energy expended correlate with one another simply because both variables also correlate with time (Halsey, 2017). This is especially a problem for diving animals when oxygen consumption is estimated after a dive of variable duration: ‘Considering flipper beats as the energy expenditure proxy provides a clear image of the mistake made in interpreting cumulative values as evidence for a relationship between rates. The seal's flippers are somewhat analogous to a ticking clock; tick‐tock, tick‐tock—the beats of the flippers count the accumulation of passing time (Halsey, 2017)’. Halsey's Time Trap can be avoided by scaling both DBA and energy expenditure to a constant time, usually a day (i.e. DEE).

DBA‐DEE validations can also inaccurately represent the goodness‐of‐fit if they fall into a ‘Mass Trap’ (Elliott, 2016). We are taught to avoid mass‐specific DEE (DEE_ms_) because the relationship between metabolism and mass is not 1:1. However, based on biomechanics (not allometry), DBA should correlate with DEE_ms_ because DBA at a constant speed should be proportional to W/m (Equation 1). Some authors express DEE as a residual on body mass, which can erode the relationship (Menzies, 2021). Others correlate DEE_ms_ with mass‐specific DBA, which inflates relationships as both variables are correlated with mass (Jeanniard‐du‐Dot et al., 2017).

Validations of DBA‐based estimates against DEE can be misleading because average DBA across a time interval may reflect the proportion of time spent in different activities, rather than predicting DEE within each activity (Hicks et al., 2017; Stothart et al., 2016). For example, if an animal switches between two behaviours with distinct average DBA and DEE values, a correlation will emerge simply because individuals differ in how much time they spend in each behaviour. This is conceptually like the cut‐point METs used in human studies. To disentangle this effect, studies compare DBA models with time budget models. Some wildlife studies find that time budget models outperform DBA models (cut‐point METs are preferred; Tremblay et al., 2024), while others find that DBA models outperform time budget models (regression METs are preferred; Stothart et al., 2016), although it is unclear which conditions favour each approach.

Other factors influencing the DBA‐DEE relationship are environment, DBA calculation, tag location and DEE method (Wilson et al., 2020). Environment can alter DBA‐DEE relationships by changing DBA without altering DEE (e.g. wave action that adds external energy without influencing energy costs of the animal), changing DEE without altering DBA (e.g. thermoregulation altering energy costs of the animal without altering DBA unless shivering or heat substitution by activity occurs) or changing both (walking on sand dunes increases energy costs despite lower acceleration). Some studies calculate VeDBA‐DEE relationships and others ODBA‐DEE, but it is unclear which one is generally preferable, although VeDBA is less impacted by tag orientation (Qasem et al., 2012; but see Wilson et al., 2020). Likewise, tag location (neck, tail, back) or secureness (ability to rotate) can vary based on study species or season, and although tag location and secureness clearly impact DBA, it is unclear whether those parameters impact the ability of DBA to predict DEE (Wilson et al., 2020). Finally, some studies use the single‐sample DLW method and others the two‐sample DLW method, with the latter possibly increasing stress and thus DEE independent of DBA (Schultner et al., 2010).

Heart rate (*f*_*H*_) is an alternative to DLW for validating DBA for species that cannot be recaptured and for timescales shorter or longer than a few days. According to Fick's principle, oxygen use (*VO*_*2*_) depends on *f*_*H*_, stroke volume (*SV*), and the difference in oxygen content between arteries and veins (*C*_*a*_ – *C*_*v*_):

(2) $${\mathrm{VO}}_{2}=f_{H}\;\mathrm{SV}\;\left(C_{a}-C_{v}\right)$$

Over a set time, this provides an equation for work, which is proportional to DBA (Equation 1). If *SV* is proportional to (heart) mass, then *f*_*H*_ is proportional to DBA for a constant speed, time and oxygen difference. However, a sprinter has high *f*_*H*_ for several minutes after sprinting stops, climbing a hill increases *f*_*H*_ but lowers acceleration compared to walking downhill, gannets have high *f*_*H*_ during glides between flaps (Ropert‐Coudert et al., 2006), and elephant seals have low *f*_*H*_ while diving even when active (Williams & Ponganis, 2021), illustrating that *f*_*H*_ and DBA can become decoupled over short time scales (seconds to minutes). When averaged over longer time scales (10 min or more), DBA and *f*_*H*_ are strongly correlated in many wild animals, within and across activities (Duriez et al., 2014; Green et al., 2009; Hicks et al., 2017). Although this relationship occurs weakly even at rest (Green et al., 2009 [*R* ^2^ = 0.22]; Hicks et al., 2017 [*R* ^2^ = 0.32]), it is strongest during very active periods when the noise associated with non‐active costs is small (Bishop et al., 2015 [*R* ^2^ = 0.91]; Weimerskirch et al., 2016 [*R* ^2^ = 0.76]).

I review all studies using accelerometry to measure energy expenditure in free‐living animals, focusing on those that calibrate DBA against DLW or *f*_*H*_. First, I examine trends (country, date, metric, taxon, topic) in accelerometer‐based energetics. Next, this review tests the overarching hypothesis that wild animal metabolism (DEE) is determined by mechanical work, as approximated by DBA according to Newton's Second Law. I predict that (i) DBA should correlate more strongly with DEE_ms_ than raw DEE (as predicted by Equation 1), (ii) DEE_ms_ should correlate more strongly with VeDBA than ODBA (as it is less sensitive to tag position) and (iii) activity‐specific models for DEE_ms_ that include DBA should outperform both average DBA models and time budget models (as the DBA‐DEE relationship is likely activity‐specific). I also examine the role of environment (where multiple studies occur on a species), species, tag location and DLW method on the DBA‐DEE_ms_ relationship. Using six seabird species with similar activity modes, I examine the potential for a general DBA‐DEE_ms_ model across species without costly and challenging calibrations.

## 2 METHODS

In April 2025, I collated all studies using accelerometers to directly measure energy expenditure in the wild (as described in Supporting Information). I then collated DBA‐DEE relationships where DEE is estimated via DLW or heart rate in free‐living animals (wild animals or farmed/captive animals in wild‐like conditions). To examine predictions (i) and (ii), I plotted the relationship between both DEE and DEE_ms_ and both ODBA and VeDBA (or whichever was available). I used t‐tests and univariate regressions to examine whether the R^2^ value of the DBA‐DEE_ms_ relationship was associated with taxon, mount, DLW method, sex or average ambient temperature. To examine the prediction that activity‐specific models for DEE_ms_ that include DBA should outperform time budget models, using data from similar animals (six species of seabirds), I compare that best‐fit DBA model to both the global model and the best‐fit time budget model, where ‘best‐fit’ is lowest AIC. I then examine how the METs vary among different activities across species.

## 3 RESULTS

**FIGURE 1:**
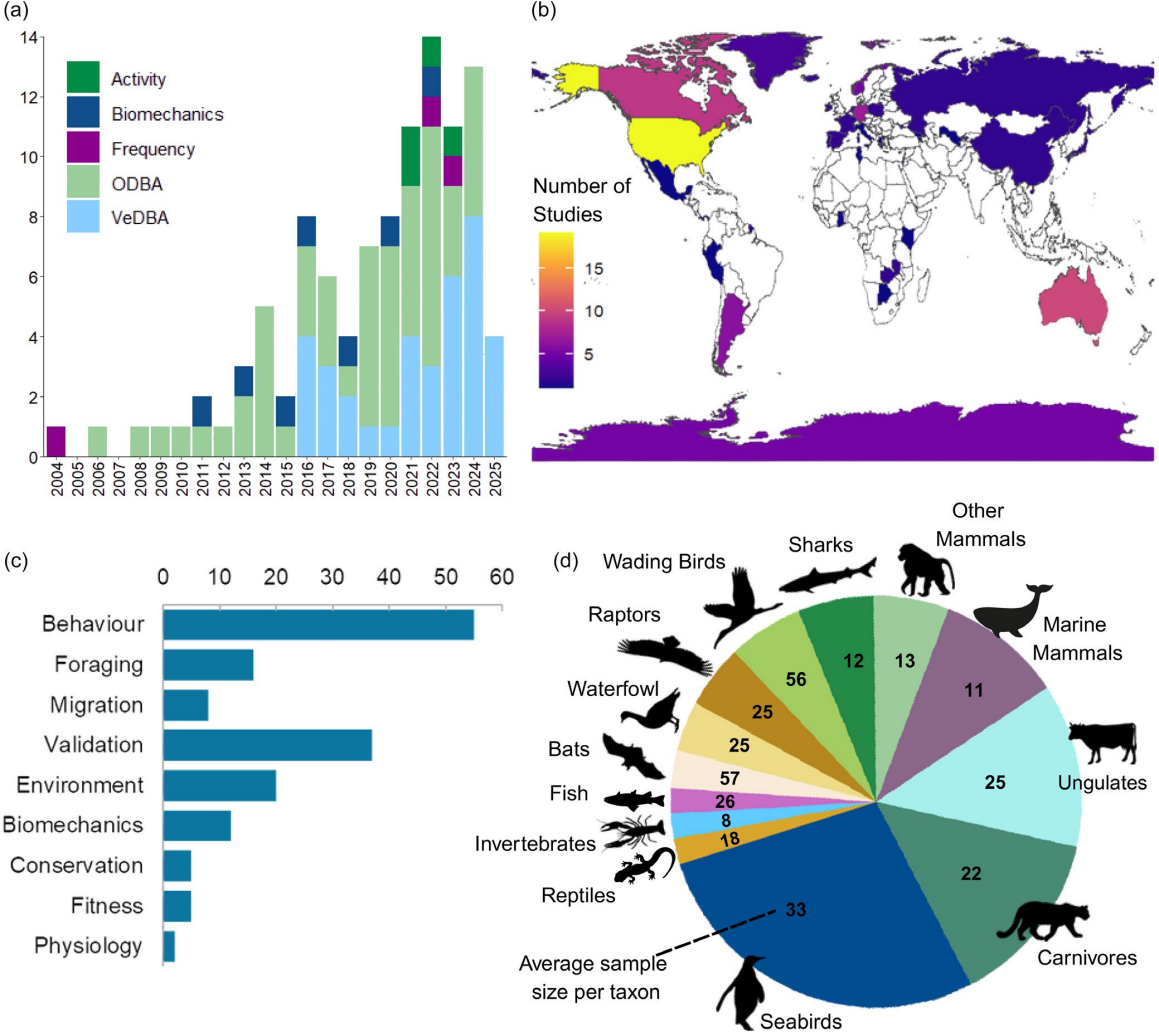
A summary of studies measuring energy expenditure in the wild using accelerometry (from Table S2). (a) Number of studies per year using each technique (ODBA, VeDBA, more complex biomechanics‐derived metrics, accelerometer‐derived ‘activity’ and tail/flipper frequency); (b) Map of location where animals were originally tagged; (c) Number of studies covering each topic (some studies covered more than one topic): Quantifying costs of behaviours (including studying foraging or migration costs which are shown separately in light blue but also included in dark blue under ‘behaviour’), validating the technique, quantifying how costs changed with environment (time, space or breeding status), testing biomechanical predictions, or associating costs with conservation & welfare, fitness or physiology; (d) Proportion of studies by taxon that report DEE measured by DBA.

The use of accelerometry to quantify energy consumption has risen rapidly, with VeDBA being the most common metric in recent years (Figure 1; Table S2). Most (91) of the 103 studies using accelerometry to quantify energy consumption were on endotherms, especially waterbirds, carnivores, ungulates and marine mammals. Sample sizes were lower for challenging taxa, such as sharks and marine mammals, than for waterbirds and bats. Most studies occurred in North America or Europe, or their overseas territories. Apart from validation studies, quantifying the costs of behaviour or the impact of the environment on costs were the most common topics studied.

**FIGURE 2:**
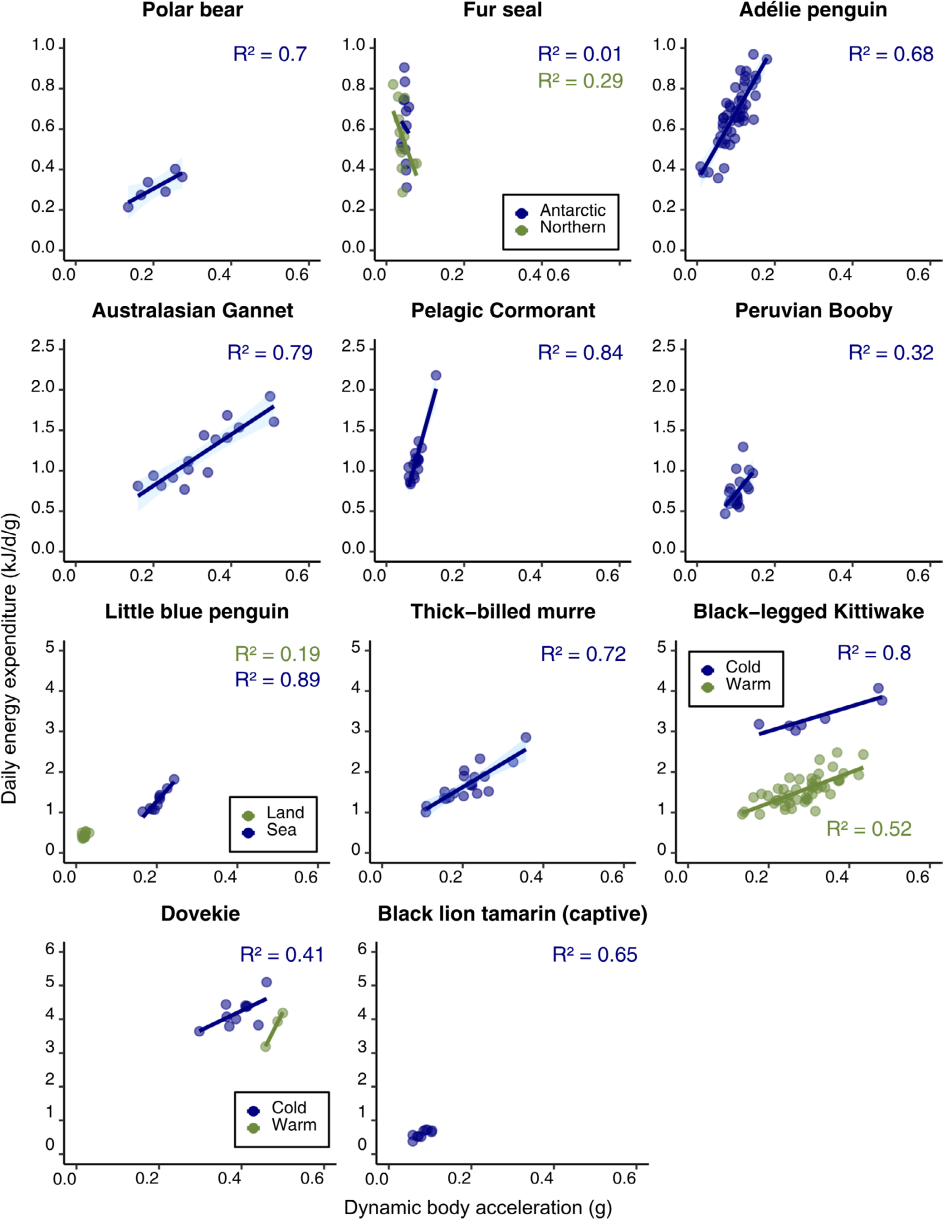
Relationship between mass‐specific daily energy expenditure estimated from doubly labelled water and dynamic body acceleration across 11 species, from largest (polar bear) to smallest (dovekie).

Out of 13 studies on 13 species (including two studies on kittiwakes and one study on two species of fur seal) that examined DBA‐DEE_ms_ relationships, all but one (fur seals) showed a statistically significant, positive relationship (Figure 2; see Table S1 for excluded studies). The two studies on kittiwakes and 2 years on dovekies had significantly different intercepts (*p* < 0.001) but not slopes (*p* > 0.05). The two species of fur seals did not differ in intercept or slope (*p* > 0.05). One study (cormorants) had an outlier that aligned with overall DBA‐DEE_ms_ trends but that also inflated goodness‐of‐fit (*R* ^2^ value: 0.84 with outlier, 0.54 without outlier). The goodness‐of‐fit increased with variability in DBA; those studies with low *R* ^2^ (seals, boobies) had little variation in DBA to work with.

**FIGURE 3:**
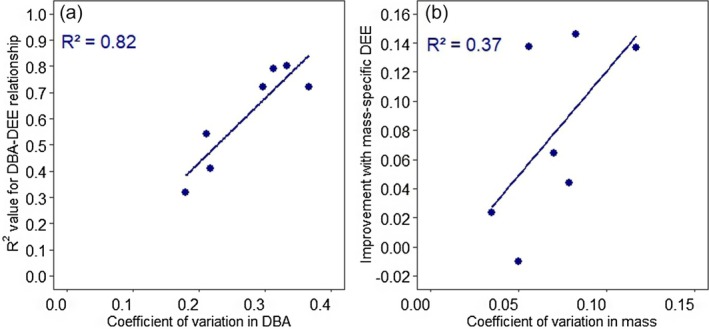
Relationships explaining variation in dynamic body acceleration–daily energy expenditure (DBA‐DEE) relationships across species of seabirds. (a) *R* ^2^ value for the DBA‐DEE_ms_ relationship depends on the coefficient of variation in DBA. (b) Improvement in the *R* ^2^ value for the DBA‐DEE relationship with DEE_ms_ compared with total DEE depends on the coefficient of variation in body mass.

There was no association between the goodness‐of‐fit (*R* ^2^ value) for DBA‐DEE_ms_ relationships and sex, ambient temperature, DLW method or mounting position of the tag (*p* > 0.05; Table 1). Agreeing with hypothesis (i), DEE_ms_ outperformed raw DEE in 10 out of 11 species (*z* = 2.73, *p* = 0.01; Table 1). The goodness‐of‐fit was higher in studies with more variable body mass (Figure 3). Agreeing with hypothesis (ii), VeDBA outperformed ODBA in all six DLW studies to date that compared the fit, but the improvement in fit was marginal (difference in *R* ^2^ between VeDBA and ODBA: Adelie penguin: 0.0009; murre: 0.0017; cormorant: 0.0035, dovekie: 0.0033; booby: 0.0056; kittiwake: 0.0018).

**TABLE 1:** *R* ^2^ value for the relationship between daily energy expenditure (DEE; estimated from doubly labelled water) and dynamic body acceleration (DBA) for mass‐specific (kJ/d/g) and raw DEE (kJ/d; negative relationships are shown in italics), and the difference (‘Difference’) between mass‐specific and raw DEE. I report the mounting of the device as neck collar (‘Neck’), taped to the back (‘Back’) or taped to the tail feathers (‘Tail’), the sex of individuals in the study (M = Male, F = Female, B = Both), the dominant activity when not at rest (A = Arboreal, F = Flying, S = Swimming, W = Walking), the sample size (“N”), the DLW method (S = single sample, T = two sample) and the DBA metric used (V = VeDBA, O = ODBA, P = partial dynamic body acceleration [ODBA in *x* and *z* planes]; A = Activity). Studies are from free‐living animals (wild, farmed or captive). See Table S1 for excluded studies.

Species	Mount	Sex	Mode	*N*	DLW	DBA	*R* ^2^ (kJ/d/g)	*R* ^2^ (kJ/d)	Difference	References
Australasian gannet	Tail	B	SF	15	T^a^	V	0.79	0.75 (0.75)	0.04	Sutton et al. (2023)
Peruvian booby	Tail	B	SF	21	S	V	0.32	0.19 (0.15)	0.13	van Oordt et al. (2024)
Pelagic cormorant	Tail	M	SF	17	S	V^b^	0.84	0.81 (0.83)	0.03	Stothart et al. (2016)
Kittiwake (Alaska)	Tail	M	F	49	T^a^	V	0.52	0.45 (0.50)	0.07	Tremblay et al. (2024)^f^
Kittiwake (Svalbard)	Back	B	F	8	T	O	0.80	0.35 (0.01)	0.45	Kristiansen (2014)^f^
Dovekie	Back	B	SF	13	S	O	0.41	0.27 (0.38)	0.14	Ste‐Marie et al. (2022)^g^
Thick‐billed murre	Back	B	SF	21	T^a^	P^b^	0.72	0.73 (0.72)	−0.01	Elliott et al. (2013)
Adelie penguin	Back	B	S	48	T	V^b^	0.68	0.71 (0.71)	0.01	Hicks et al. (2020)
Little blue penguin	Back	B	S	22	T^a^	V	0.89/0.94^d^	0.70/0.91^d^ (0.84/0.91)	0.19/0.03^d^	Sutton et al. (2021)
Black lion tamarin^h^	Back	B	A	10	T	V	0.65	0.46 (0.67)	0.19	Rezende et al. (2023)
Northern fur seal	Back	F	S	12	T	V^b^	0.00^e^			Jeanniard‐du‐Dot et al. (2017)
Antarctic fur seal	Back	F	S	13	T	V^b^	0.33^e^			Jeanniard‐du‐Dot et al. (2017)
Red squirrel	Neck	B	W	16	T	O		(0.03)		Menzies (2021)
Canada lynx	Neck	B	W	8	T	O		(*0.55*)		Menzies (2021)
Snowshoe hare	Neck	B	W	23	T	O		(*0.08*)		Menzies (2021)
Polar bear	Neck	F	SW	6	T	O	0.70	0.62 (0.23)	0.08	Pagano and Williams (2019)
Reindeer	Neck	F	W	14	T	A^c^	0.26			Trondrud, Pigeon, Król, et al. (2021)

^a^ Birds freed after injection and recaptured for the initial blood sample, minimizing capture stress with a two‐sample method. For murres and kittiwakes (Alaska), those individuals not recaught were estimated via the single‐sample method.

^b^ Examined correlation between kJ/d/g and both VeDBA and ODBA/PDBA, and selected VeDBA as it had a higher correlation.

^c^ The difference in acceleration between two consecutive measurements, characterising the mean acceleration in each axis over a 5‐min period within a relative range between 0 and 255. X and Y were summed over the DLW measurement period and divided by the duration of this period (in days).

^d^ Reports the relationship between DBA/kg and DEE/kg.

^e^ Reports values separately for birds that went to sea (presented here as being more comparable to other studies) and those that stayed at the colony; the combined values for birds that stayed at the colony and the sea are shown after the slash.

^f^ Sea surface temperature at Middleton Island, Alaska in June 2021 (during experiments) was 12°C. Sea surface temperature in Kongsfjorden, Svalbard in July 2012 was 5°C. Alaska birds were pre‐laying and incubating while Svalbard birds were chick‐rearing.

^g^ Sea surface temperature was 1.7°C in 2018 and 0.7°C in 2017. The *R* ^2^ is only shown for 2017 due to the small sample size (*N* = 3) in 2018.

^h^ Captive study.

The relationship between *f*_*H*_ and DBA was consistently higher within than across individuals for those studies that examined both, with R^2^ improving on average by 0.27 for species where both were compared. The relationship was strong for all species except terrestrial mammals in winter (Menzies, 2021; Trondrud, Pigeon, Albon, et al., 2021; Kirchner, 2024) or species where *f*_*H*_ and DBA values were averaged over 1 min or shorter (e.g. Kirchner, 2024; Niccolai et al., 2024). Beyond that time scale, there was no consistent trend for the relationship to improve with time (Table 2).

**TABLE 2:** *R* ^2^ value for the relationship between heart rate and dynamic body acceleration (DBA) from free‐living animals (wild unless described as ‘Captive’). I report the dominant mode when not at rest (F = Flying, S = Swimming, W = Walking), the sample size (“N”), the DBA metric used (V = VeDBA, O = ODBA, A = Activity, P = partial dynamic body acceleration in *z*‐axis (P)), and the state when the measurements took place.

Species	Mode	DBA	State	*N*	Time scale	*R* ^2^ (within individuals)	*R* ^2^ (pooled)	References
Canada lynx	W	O	All	8	1 day		0.05	Menzies (2021)
Canada lynx	W	O	All	8	1 h		0.56	Menzies (2021)
Canada lynx	W	O	All	8	10 min		0.12	Menzies (2021)
Snowshoe hare	W	O	All	23	1 day		0.00	Menzies (2021)
Snowshoe hare	W	O	All	23	1 h		0.04	Menzies (2021)
Snowshoe hare	W	O	All	23	10 min		0.03	Menzies (2021)
Red squirrel	W	O	All	16	1 day		0.18	Menzies (2021)
Red squirrel	W	O	All	16	1 h		0.33	Menzies (2021)
Red squirrel	W	O	All	16	10 min		0.19	Menzies (2021)
Himalayan griffon	F	O	Flight	1	1 min	0.73		Duriez et al. (2014)
Eurasian griffon	F	O	Flight	1	1 min	0.69		Duriez et al. (2014)
Bar‐headed goose	F	P	Flight	1^c^	1 h	0.91		Bishop et al. (2015)
European shag	SF	O	All	12	1 min^d^		0.38^b^	Hicks et al. (2017)
European shag	SF	O	All	12	1 day		0.97^b^	Hicks et al. (2017)
Lesser black‐backed gull	F	V	All	6	5 min		0.74	Brown et al. (2022)
Black‐browed/grey‐headed albatross	F	O	Flight	14	1 day	0.78^e^	0.25^e^	Conners et al. (2024)
Black‐browed/grey‐headed albatross	F	O	Flight	14	12 h	0.72^e^	0.26^e^	Conners et al. (2024)
Black‐browed/grey‐headed albatross	F	O	Flight	14	30 min	0.51^e^	0.16^e^	Conners et al. (2024)
Moose	W	O	All	8	4 s		0.05^a^	Kirchner (2024)
Northern bald ibis	F	O	Flight	4	1 min		0.61	Mizrahy‐Rewald et al. (2022)
White stork	F	O	All	20	1 day		0.38^b^	Flack et al. (2020)
Greater spear‐nosed bat	F	V	All	11	1 min		0.30^a^	Bayer (2024)
Great frigatebird	F	V	Flight	11	1 s		0.76	Weimerskirch et al. (2016)
Reindeer (Winter, resting)	W	A^f^	All	19		0.05	0.02	Trondrud, Pigeon, Albon, et al. (2021)
Reindeer (Summer, resting)	W	A^f^	All	19		0.42	0.16	Trondrud, Pigeon, Albon, et al. (2021)
Reindeer (Winter, active)	W	A^f^	All	19		0.07	0.06	Trondrud, Pigeon, Albon, et al. (2021)
Reindeer (Summer, active)	W	A^f^	All	19		0.38	0.24	Trondrud, Pigeon, Albon, et al. (2021)
Cattle (Captive)	W	O	All	14	1 min		0.13^a^	Niccolai et al. (2024)
Oryx (Captive)	W	A	All	8	2 min		0.43	Leimgruber et al. (2023)
Cattle (Captive)	W	O	All	8	10 min	0.64	0.51	Miwa et al. (2015)
Cattle (Captive)	W	O	All	8	1 day	0.53	0.37	Miwa et al. (2015)
Goat (Captive)	W	O	All	6	10 min	0.65	0.46	Miwa et al. (2015)
Goat (Captive)	W	O	All	6	1 day	0.70	0.74	Miwa et al. (2015)
Sheep (Captive)	W	O	All	5	10 min	0.68	0.54	Miwa et al. (2015)
Sheep (Captive)	W	O	All	5	1 day	0.93	0.42	Miwa et al. (2015)

^a^ *R* ^2^ from general additive mixed model.

^b^ Marginal *R* ^2^ from multiple regression including other factors.

^c^ Although data for 4 birds is available, this regression is presented in the manuscript for only one bird.

^d^ Values calculated over each ‘behavioural state’ (i.e. over an entire behaviour before a switch in state), which for diving and flying was typically on the order of minutes.

^e^ Individual = conditional *R* ^2^; pooled = marginal *R* ^2^.

^f^ Accelerometer used to determine whether animal is above 1.6 km/h (‘active’).

**FIGURE 4:**
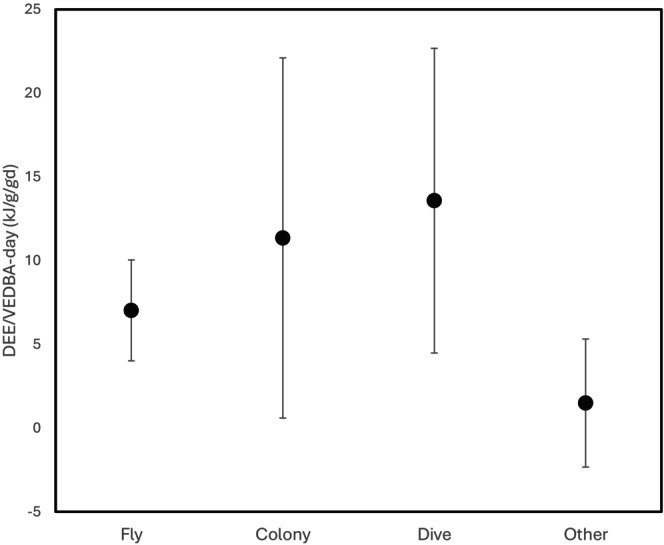
Average coefficients relating VeDBA to mass‐specific daily energy expenditure (DEE) across six species of seabirds, for each of four different activity types. Only activities that were not pooled with other activities in the full model are shown. All activities, including those classified as “Other” are shown in Table 2.

The activities used for time‐energy budgets varied among studies (Table 3). The activity‐specific model always outperformed the null (all activities pooled), even if sometimes the model was more parsimonious if some activities were pooled together (Table 3). Thus, the DBA‐DEE_ms_ relationship varied across activities. Agreeing with hypothesis (iii), the DBA model (Equation 3 in Detailed Methods) outperformed the time budget model (Equation 2) in every study, illustrating that DBA contributed to the prediction of DEE within activities and not just because it was a good proxy of activity (Table 3). The DBA‐DEE_ms_ coefficients (METs) were highly variable across studies for the same activity, except possibly for flight (Figure 4).

**TABLE 3:** Activity‐specific models for the relationship between DEE_m_ (from DLW in kJ/d/g) and DBA in six seabird species. The ‘Pooled’ ΔAIC value is shown for the best‐fit (lowest AIC) model relative to the model without any activities (“Pooled”). The “Time” ΔAIC is shown for that same model compared to the best‐fit (lowest AIC) time budget model.

Species	Global model	Best‐fit model	*R* ^2^	ΔAIC (pooled)	ΔAIC (time)	References
Kittiwake	Flap + Soar + Swim + Colony + Rest	0.80 + 3.48*Flap +4.80*Swim^c^	0.55	3.45	1.74	Tremblay et al. (2024)
Peruvian booby	Fly + Rest + Colony	−0.23 + 6.50*Fly +14.7*Colony^c^	0.42	1.03	3.20	van Oordt et al. (2024)
Pelagic cormorant	Fly + Dive + Swim + Colony	0.36 + 19.3*DiveFly +2.4*SwimColony	0.88	3.73	8.08	Stothart et al. (2016)
Dovekie^a^	Fly + Dive + Other + Year	1.56 + 11.1*Fly +9.9*Dive −0.43*Year2018^c^	0.90	18.63	8.31	Ste‐Marie et al. (2022)
Thick‐billed murre^b^	Fly + Dive + Swim + Colony	0.15 + 6.91*Fly +6.85*Swim +22.4*DiveColony	0.80	9.98	4.39	Elliott et al. (2013)
Adelie penguin	Porpoise + Dive + Surface + Preen + LandPreen + Rest + Walk + Sex	0.46–2.99*Preen +1.78*PorpoiseDiveSurface − 0.91*LandpreenRestWalk − 0.03*SexM	0.75	22.23	5.09	Hicks et al. (2020)

^a^ Models recalculated from raw data as the original paper did not present models for mass‐specific DEE.

^b^ Authors deployed biaxial accelerometers and report ‘partial dynamic body acceleration’ representing ODBA on two axes.

^c^ The best DBA model with all parameters is Kittiwake: 0.81 + 3.66*FlapSwim −0.02*SoarColonyRest (ΔAIC = 2.00 from best‐fit model); Boobies: −0.26 + 6.01*FlyRest + 15.6*Colony (ΔAIC = 0.09); Dovekies: 1.51 − 0.09*Other + 10.74*FlyDive − 0.39*Year2018 (ΔAIC = 0.37).

## 4 DISCUSSION

Accelerometry, especially with VeDBA as a metric, is increasingly used to estimate energy expenditure in wild animals, at least in wealthy countries (Figure 1). Nonetheless, the approach is mostly restricted to a few taxa (large birds and mammals) where it has been validated in similar taxa (Figure 1). There are few studies on ectotherms (Figure 1), even though relationships should be stronger due to low non‐active metabolism. Encouragingly for homeotherms, DBA was an accurate and linear proxy for DEE_ms_ in 10 out of 11 DLW studies and nine out of nine within‐individual heart rate studies (but reindeer only in summer) (Figure 2), providing a consensus for the overarching hypothesis that simple Newtonian biomechanics describes whole organism metabolism across a wide variety of environments and taxa. The only exception in Figure 1 was a study on fur seals where variation in DBA was low. Relationships were also weak for terrestrial mammals in winter, when running in snow has low DBA but high energy costs and thermoregulation is high, and for ruminants where digestive costs are high (Tables 1 and 2). The use of DBA to predict DEE_ms_ was improved in all studies by activity‐specific coefficients implying that the DBA‐DEE_ms_ coefficients differ among activities (Table 3), likely including running in snow and digestion.

The relationship between *f*_*H*_ and DBA was variable, partly because of variable timescales and the relative number of individuals relative to measurements (Table 2). The *R* ^2^ was consistently higher within than across individuals, meaning that studies with few individuals relative to the number of measurements could have a higher R^2^. Relationships were weak at short timescales (1 min or shorter), but, excepting terrestrial animals in winter, relationships were strong at longer timescales. Other causes of variability may have been methodological. DBA and *f*_*H*_ were usually measured by different tags with different clocks, meaning that drift needed to be accounted for. Studies usually assumed a linear drift when drift may depend on temperature. Also, many studies that measured *f*_*H*_ at longer (e.g. 10 min) intervals did so by measuring *f*_*H*_ for only 1–2 s every interval, which is essentially the same as using a 1‐s scale (e.g. Brown et al., 2022). Alternatively, some studies compared those 1‐s measurements to DBA averaged over long timescales creating similar issues (e.g. Trondrud, Pigeon, Albon, et al., 2021).

In all studies but one (murres, where variation in mass was low), DBA predicted DEE_ms_ better than raw DEE or residual DEE (Table 1; Hypothesis i). Furthermore, all studies that examined the relationship between DEE_ms_ and ODBA or VeDBA found a very small but consistent improvement in fit with VeDBA. ODBA should more accurately represent muscle energy output if accelerometers are placed in line with body (and muscle) motion (Wilson et al., 2020) and outperforms VeDBA in the laboratory (Bidder et al., 2017; Qasem et al., 2012). In the wild, where tag placement and stability are likely more problematic, it seems that VeDBA may perform marginally better as it is less sensitive to variation in tag orientation—confirming Hypothesis ii. Fortunately, ODBA and VeDBA are closely correlated and can be converted to one another via the formula ODBA = 1.44 VeDBA (R^2^ = 0.998; Wilson et al., 2020). To provide comparability with the growing consensus towards using VeDBA (Table 1, Figure 1), I recommend that studies use VeDBA to predict DEE_ms_.

Goodness‐of‐fit of the DBA‐DEE_ms_ relationship was only explained by variation in DBA; studies with high variation in DBA had high *R* ^2^ values. A study with 50% coefficient of variation in DBA would have nearly 100% variation in DEE_ms_ explained (Figure 2)—remarkable given DLW is only accurate to 20% among individuals and so measurement error should be significant (Speakman, 1997).

### 4.1 Avoiding the time trap, mass trap and issues of collinearity

The approach used here avoids Halsey's Time Trap as all values are scaled to a single day. The coefficients in Equation 3 can be scaled up to longer periods or down to shorter periods, such as per second to obtain kW (kJ/s). Sutton et al. (2021) used an innovative alternative approach by subtracting the energy cost of being at the colony based on individuals that stayed at the colony for the entire period. However, this approach does not work for species that do not stay at the colony for days (e.g. Sutton et al., 2023) and does not allow calculation of activity‐specific DEE‐DBA relationships for activities away from the colony. Sutton et al. (2021) and Sutton et al. (2023) calculated activity‐specific coefficients by using time budget models to calculate activity‐specific costs per unit time and dividing those by average VeDBA, essentially applying the MET cutoff method. However, this does not consider that VeDBA may be related to energy expenditure within an activity.

DBA was related to DEE_ms_ as expected from biomechanics. The DBA‐DEE_ms_ relationships can also be inflated by a ‘mass trap’ because large variation in mass can lead to a high R^2^ value (as both DEE_ms_ and DBA are negatively associated with body mass) even if DEE and DBA are not related at a given body mass. The ‘Mass Trap’ can be overcome in validation experiments by showing the relative contribution statistically of body mass and DBA on DEE, or by using individuals that do not vary much in body mass. While the DBA‐ DEE_ms_ relationship should be linear for the mechanical component of DEE, the resting component (and the metabolic efficiency of active components) of DEE should increase allometrically, and future studies using *f*_*H*_ might be able to separate resting (allometric) from mechanical (inverse) relationships between DEE and mass.

We are also taught to avoid collinearity in general linear models, yet this is often impossible in activity‐specific DBA‐DEE_ms_ analyses as time in each activity (and thus integrated DBA per day) is necessarily correlated with time in other activities (Wilson & Culik, 1993). Time spent moving per day must be negatively correlated with time spent resting if only those two activities are considered, while time spent in one activity (i.e. diving) is likely to be correlated with time spent in another activity (i.e. flying) as birds on distant, longer trips spend more time foraging. Thus, even if DBA during diving does not actually relate to energy expended during diving, the model may consider diving a significant predictor of DEE_ms_ if it is correlated with flying and flying is a significant predictor of DEE_ms_, inflating the coefficient for diving and deflating the coefficient for flying (Wilson & Culik, 1993). Solutions include pooling activities that are strongly correlated with one another, using multivariate approaches (e.g. PCA), or at least considering how collinearity influences the coefficients assigned to each activity. Unrealistic coefficients, such as negative coefficients or coefficients very different from those estimated in other species, should be examined with circumspection.

### 4.2 DBA‐DEE_ms_ relationships are not transferable across contexts

The slope of the DBA‐DEE_ms_ relationship (the METs), representing the efficiency that mechanical work (approximated by DBA) is converted into metabolic work (approximated by DLW), varies among environmental conditions within a species (Table 1) but not among two seal species in different environments (Jeanniard‐du‐Dot et al., 2017). Not surprisingly, small birds diving (dovekies) or sitting (kittiwakes) in colder water have higher DEE_ms_ for a given DBA (Figure 2; Table 1) as they must expend extra energy to thermoregulate in cold waters, even if higher activity can sometimes substitute for thermal costs. Moreover, chick‐rearing kittiwakes in Svalbard had higher DEE_ms_ for a given DBA than incubating and pre‐laying kittiwakes in Alaska, perhaps because they flew more in Alaska where the foraging range is much larger than in Svalbard. The increase in DEE per DBA (~1 kJ/d/g in dovekies; ~2 kJ/d/g in kittiwakes) was more than 10 times higher than that expected based on the given difference in air or water temperature (~0.05 kJ/d/g in dovekies [conductivity = 0.063 mLO_2_/gh°C]; ~0.2 kJ/d/g in kittiwakes [conductivity = 0.047 mLO_2_/gh°C]), showing that the discrepancy is not due to thermoregulation alone. Clearly, DBA‐DEE_ms_ relationships derived from one environment cannot be easily translated to another environment.

Given that DBA has been validated extensively on treadmills (Halsey et al., 2009, 2011; Wilson et al., 2006), it is surprising that some of the poorest calibrations come from terrestrial mammals (Menzies, 2021; Kirchner, 2024; see general patterns in Table 2). I propose three explanations. First, many species in Table 2 are ruminants, which are inactive (compared to, say, a mustelid) with high digestive costs. Indeed, in farm situations, researchers often tag cattle with multiple accelerometers (ear, leg, neck) to quantify rumination (e.g. Benaissa et al., 2019), which future researchers might consider for wild ruminants. Second, many of the mammalian studies occurred across seasons because large mammals can wear large tags with large batteries, and thermoregulatory costs (including changes in thermal conductivity due to winter fur or changes in resting metabolism due to lower digestive organ size) vary across seasons, obscuring relationships (Menzies, 2021). Third, whereas air and water are relatively homogenous, land is heterogeneous. Running on sand, heavy brush or snow reduces peak acceleration per stroke and thus DBA while increasing DEE, confounding relationships (Halsey et al., 2011; Wilson et al., 2020). Future work could calibrate DBA on different substrates. There are many other conditions that could alter relationships by increasing the contribution of resting metabolism relative to active metabolism, such as hibernation/torpor, overheating, reproduction/gestation/growth and digestion. Likely, DBA will always be challenging for species with very low metabolism (seals during dives, koalas and sloths, etc.). Nonetheless, the development of calibrations in different environments should be possible and could lead to a general model as seems to be possible in flight (see Section 4.3), with separate coefficients for running on different substrates or with a term for thermoregulation.

It is tempting to consider the intercept as equivalent to resting metabolic rate; that is, energy expenditure with no DBA. This is the case for animals running on treadmills where the lowest (or no) speed must necessarily be positive and equal to ‘rest’ (whatever that is for an animal in experimental conditions) (Wilson et al., 2020). However, there was no relationship among species between the intercept and resting metabolic rate. For example, the actual mass‐specific resting metabolic rate (RMR) in murres (intercept = 0.15 kJ/d/g; RMR = 6.9 W/kg from McKechnie & Wolf, 2004) is considerably higher than that of the Adelie penguin even though the intercept for penguins is higher (0.46 kJ/d/g; RMR = 3.1 W/kg from McKechnie & Wolf, 2004). In boobies, the intercept is negative (Table 3). Most other species in Table 3 do not have known RMR values. Rather than representing rest, the intercept may be a statistical artefact representing error (see also Wilson & Culik, 1995).

### 4.3 Towards a general physiological model

There is an urgent need for a general model relating VeDBA to DEE_ms_ as many researchers are unlikely to calibrate VeDBA directly (e.g. Chakravarty et al., 2023). The smaller error bars on the flight coefficients (Figure 4) hint at a way forward that could be generalized beyond seabirds. These coefficients or METs are inversely related to the efficiency at which muscles convert oxygen (DEE) to useful work (DBA), which is typically 0.23 across species during flight (Pennycuick, 2008). It is unsurprising that coefficients are highly variable for other activities that represent many non‐homogeneous activities (‘colony’ includes preening, sleeping, fighting) and are influenced by non‐active energy expenditure (thermoregulation and dive response) (Halsey, 2017; Laich et al., 2011; Wilson et al., 2020). Flapping flight coefficients are relatively consistent presumably because flight has high activity costs compared to other metabolic components (thermoregulation, resting metabolism), and so variation due to those components is likely low. If we can develop more estimates of efficiency for relatively homogenous activities, such as flight (or running at a constant gait across stable ground, surface swimming, etc.), then we should be able to apply those coefficients generally across contexts—such as the cost of digestion in ruminants, running in snow or ascending a hill (or in air). Sample size plays an important role in determining activity‐specific coefficients; a study with four activities and 20 individuals only has four degrees of freedom per activity in the global model, and five points is low to estimate the slope of a regression. The study with 48 individuals (Adelie penguins) was one of only two able to determine coefficients for three activities (Table 3). Sample size for DEE‐DBA studies is constrained because large species are expensive for DLW yet are most amenable to biologgers; hopefully, the continued miniaturization of accelerometers will open avenues to testing these relationships in taxa where costs to purchase DLW are minimal.

## 5 CONCLUSIONS

**FIGURE 5:**
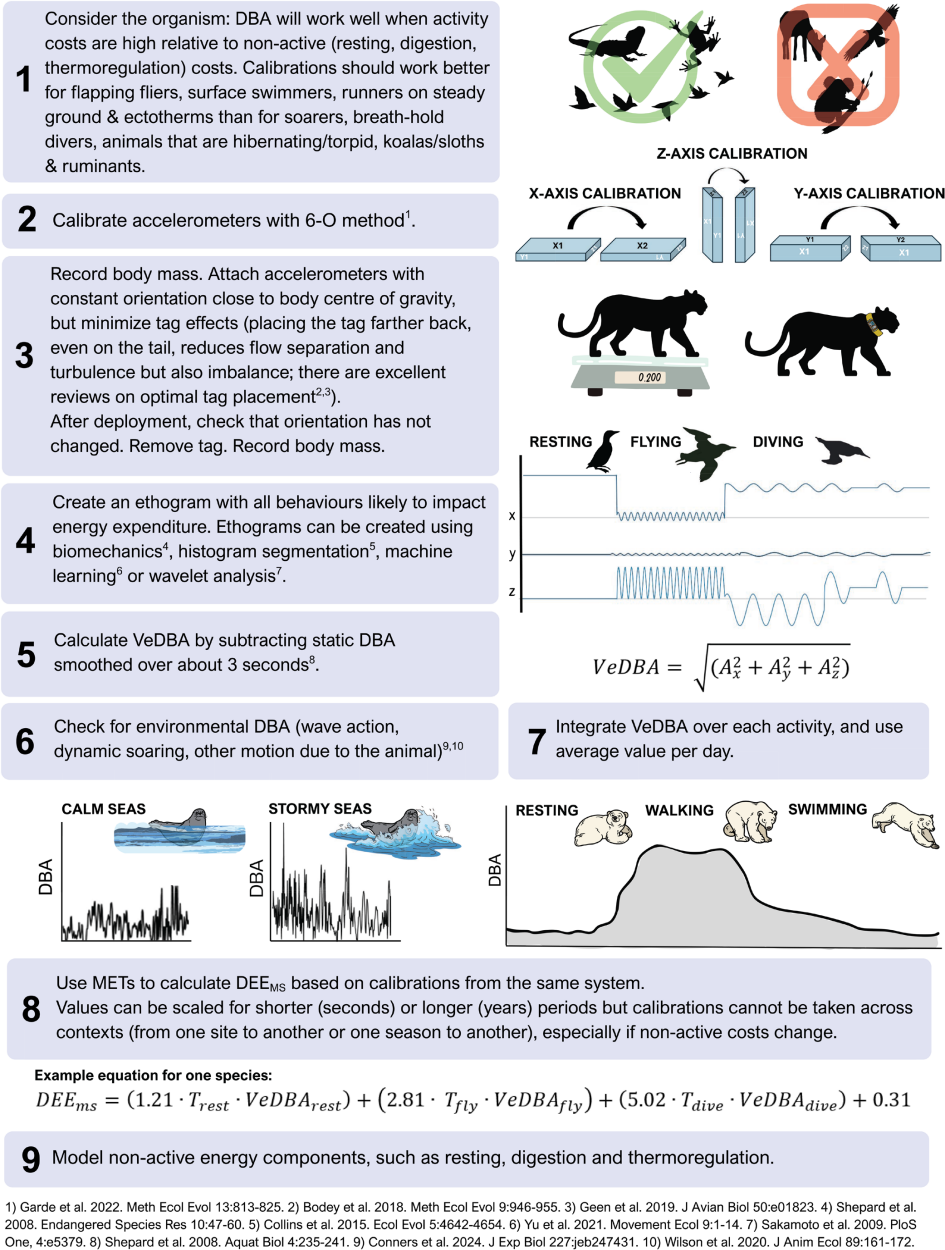
How to calibrate dynamic body acceleration (DBA) using mass‐specific daily energy expenditure (DEE_ms_), based on best practices outlined in this review.

Future studies should calibrate VeDBA against DEE_ms_ with sufficient statistical power to accurately estimate activity‐specific coefficients across many activities (Figure 5). To that end, using DLW, heart rate telemetry and DBA together would be especially rewarding. The heart rate–DBA calibration could estimate relative costs across activities, even short activities whose costs are difficult to estimate via DLW, while DLW could act as a “check” and an estimate of absolute DEE, which can be challenging to estimate via heart rate. Body temperature measurements could be helpful to calibrate energetic costs of activities, especially for animals using heterothermy. Regardless, given that the equation used to estimate DEE from DLW can cause differences in estimated DEE of up to two‐fold (Shaffer, 2011), I recommend archiving DLW measurements alongside papers so that the same equation can be applied to all datasets. Additionally, the DLW calculations typically use a constant respiratory quotient (often ~0.85), yet the respiratory quotient should vary across activities as they likely metabolize substrates in different proportions, and it would be helpful in the future to consider how variable respiratory quotients impact estimates of METs (Shaffer, 2011; Speakman, 1997). Double‐tagging to understand the impact of tag location on DBA would be helpful, as would studying the impact of temperature, wave action, and other environmental variables on the DBA‐DEE_ms_ relationship (Wilson et al., 2020).

Given the growing number of studies on seabirds, this is likely a fruitful group for additional calibrations; if a general model could be developed for this group, it would be an excellent step towards developing a general model for all species. I recommend calibrating across a range of body masses (e.g. five species from the same family varying by an order of magnitude in mass), across species using multiple flight modes (e.g. flap‐gliding, dynamic soaring) and across multiple phylogenetic groups (e.g. diving‐petrels to compare with auks). Non‐diving seabirds may be particularly helpful because of the challenges in modelling non‐activity costs associated with the dive responses and thermoregulation during diving (Halsey, 2017). Of particular interest would be comparing VeDBA as a measure of flight costs to the formula proposed by Spivey and Bishop (2013).

Despite the many environmental factors that deteriorate the DBA‐DEE_ms_ relationship, mechanical work (DBA) did accurately estimate whole animal metabolic rate (DEE_ms_), at least across most of the active homeotherms considered here (Table 1). Coupled with heart rate sensors or soon‐to‐be‐developed physiological sensors (measuring dissolved oxygen, glucose, etc.), DBA may eventually become even more accurate. Accelerometry varies with muscle ultrastructure and mitochondrial efficiency, which clearly affects DBA‐DEE_ms_ relationships (Lalla et al., 2020), and future studies could examine whether variation in DBA‐DEE_ms_ coefficients is related to muscle performance, measured via ultrasound (muscle size), biopsy (ultrastructure) or Oroborus (mitochondrial efficiency). Given their low R^2^ values across activities, validations for animals moving in different types of snow (or with/without winter fur) or ruminants in different stages of digestion (or with different stomach sizes across seasons) might also be helpful—as well as in poorly represented groups such as arboreal mammals, burrowers and ectotherms. In the meantime, the use of DBA as a proxy for energy expenditure can shed light on many aspects of animal ecology (Figure 1). Applications to date have primarily been limited to measuring the cost of behaviours, especially foraging and migratory behaviours, and associations with the environment (Figure 1). The consensus on robust DBA‐DEE_ms_ relationships (Table 1) should encourage researchers to apply accelerometry to many more topics (Figure 1), such as individual fitness (Grémillet et al., 2018), conservation threats (Grunst et al., 2023) and animal welfare (Beaulieu & Masilkova, 2024).

## CONFLICT OF INTEREST STATEMENT

The author declares no conflict of interest.

## ETHICS STATEMENT

As a review article with no new data presented, this article does not present data requiring ethics board approval.

## Supporting information


**Data S1.** All DEE‐DBA datasets used in the analyses.

**Table S1.** Papers excluded from the DLW/Heart rate vs. DBA comparisons.
**Table S2.** All studies used in Figure 1 (*n* = 103).

## Data Availability

This is a review article with no new data presented.
