# Supplementary material for: Sweat the small stuff: A review of the use of accelerometers to estimate energy expenditure in wild animals
Source: J Anim Ecol. 2025 Nov 8;94(12):2362–75. doi: 10.1111/1365-2656.70162 (PMC12673247; doi:10.1111/1365-2656.70162)
Supplement: Supplementary file 2 — Table S1. Papers excluded from the DLW/Heart rate vs. DBA comparisons. Table S2. All studies used in Figure 1 (n = 103). [file JANE-94-2362-s001.docx]

**DETAILED METHODS**

In April 2025, I collated all studies using accelerometers to directly measure energy expenditure in the wild. I searched forward and backwards from Williams et al. (2014), one of the earliest papers. I also searched Google scholar using the terms “DBA energy DEE”, “DBA dynamic body acceleration energy DEE”, “DBA dynamic body acceleration energy proxy” and Web of Science using the terms “DBA energy DEE” and “DBA dynamic body acceleration energy DEE”. I also did a forward and backward search on two reviews, Halsey et al 2011 and Wilson et al. 2020. I kept studies that used DBA as a proxy for “energy expenditure”, but not as a proxy for “activity”, and only included papers that involve captivity if they then apply it to wild animals.

I then collated DBA-DEE relationships where DEE is estimated via DLW or heart rate in free-living animals. I examined all citations in SCOPUS and Google Scholar to the first such reports (all studies: Williams et al., 2004; Wilson et al., 2006; DLW: Elliott et al., 2012; *f_H_*: Duriez et al., 2014; Hicks et al., 2017), and then examining all references within and citations to those relevant citations (Tables 1 and 2; Fig. 1). I excluded the studies in Table S1. The bibliography is entirely English language papers available online. Where data were archived alongside the article, I used those data. Where data were not archived in a suitable format, I digitized the data from graphs within the manuscript. To examine predictions (i) and (ii), I plotted the relationship between both DEE and DEE_ms_ and both ODBA and VeDBA (or whichever was available). Where data were presented as m/s^2^ I divided by 9.81 to get *g*, and where presented as MJ/kg and MJ I divided by each other to get mass. I then calculated the R^2^ value for each DBA-DEE or DBA-DEE_ms_ relationship. I also calculated the coefficient of variation among individuals for body mass and mean DBA. Finally, I also used t-tests and univariate regressions to examine whether the R^2^ value of the DBA-DEE_ms_ relationship was associated with taxon, mount, DLW method, sex or average ambient temperature.

To examine how the DBA-DEE_ms_ relationship varied when activity-specific coefficients were considered, I recorded the global model considered by all studies. All studies considered at least two models:

${DEE}_{ms}=aT_{a}+bT_{b}+cT_{c}+\ldots$ (Equation 3)

${DEE}_{ms}=a{DBA}_{a}T_{a}+b{DBA}_{b}T_{b}+c{DBA}_{c}T_{c}+\ldots+ RMR$ (Equation 4)

where *DEE_ms_* is mass-specific daily energy expenditure estimated via DLW, *T_x_* is proportion of time per day in activity *x*, *DBA_x_* is mean DBA in that activity across the deployment, *RMR* is the intercept (equal to resting metabolic rate, which is DEE when no activity occurs) and the coefficients *a*, *b*, *c*, etc. are the calibration coefficients (“METs”) that are output from the general linear model described by each equation. The time budget model (Equation 3) has no intercept as no energy is expended if no time occurs while the DBA model has an intercept (RMR) because energy is expended even if DBA is zero (i.e. resting), provided in both cases that all activities are included in the model so that the percentage of time in all activities sums to 100%. The approach above avoids Halsey’s Time Trap as both energy expenditure and dynamic body acceleration are scaled to one day. The value *DBA_x_T_x_* is the integral of DBA over the entire period, normalized to one day. To examine the prediction that activity-specific models for DEE_ms_ that include DBA should outperform time budget models, I compare that best-fit DBA model (Equation 4) to both the global model and the best-fit time budget model (Equation 3), where “best-fit” is lowest AIC. I then examine how the METs vary among different activities across species.

Table S1. Papers excluded from the DLW/Heart rate vs. DBA comparisons.

| Species | Why excluded? |  |
| --- | --- | --- |
| Northern fur seal | Compared DBA and DLW to metabolic rate measured in a pool + haulout with a metabolic chamber, but did not do DBA and DLW at the same time so cannot directly compare. | Dalton et al. (2014) |
| Puma | Sample size of only two animals did not allow for a regression. | Barceló et al. (2022) |
| American lobster | Heart rate and acceleration were compared to distance travelled but not directly to each other. | Gutzler & Watson (2022) |
| Grey seal | Heart rate and acceleration were both measured, but not directly compared to each other. Acceleration was only used to create three activity states. | Shuert et al. (2020) |
| Cape Gannet | Attached heart rate loggers and accelerometers to the same individuals, but did not calculate DBA (compared heart rate in flapping to gliding flight). | Ropert-Coudert et al. (2006) |
| Meerkats | Attached accelerometers and used historical DLW relationships to estimate energy expenditure. | Chakravarty et al. (2023) |

References:

Barceló, G., Pauli, J.N., Alldredge, M., & Karasov, W.H. (2022). Assessment of behavioral energetics model on *Puma concolor* using doubly labeled water. *Canadian Journal of Zoology*, **101**, 199-206.

Chakravarty, P., Cozzi, G., Scantlebury, D.M., Ozgul, A. and Aminian, K., 2023. Combining accelerometry with allometry for estimating daily energy expenditure in joules when in-lab calibration is unavailable. *Movement ecology*, *11*(1), p.29.

Dalton, A. J. M., Rosen, D. A. S., & Trites, A. W. (2014). Season and time of day affect the ability of accelerometry and the doubly labeled water methods to measure energy expenditure in northern fur seals (*Callorhinus ursinus*). *Journal of Experimental Marine Biology and Ecology*, *452*, 125–136. https://doi.org/10.1016/j.jembe.2013.12.014

Gutzler, B. C., & Watson III, W. H. (2022). Measurements of American lobster heart rate and movements in the wild using a low-cost open source datalogger. *Marine Biology*, *169*(4), 52. https://doi.org/10.1007/s00227-022-04046-7

Ropert-Coudert, Y., Wilson, R.P., Grémillet, D., Kato, A., Lewis, S. and Ryan, P.G., 2006. Electrocardiogram recordings in free-ranging gannets reveal minimum difference in heart rate during flapping versus gliding flight. *Marine Ecology Progress Series*, *328*, pp.275-284.

Shuert, C. R., Halsey, L. G., Pomeroy, P. P., & Twiss, S. D. (2020). Energetic limits: Defining the bounds and trade-offs of successful energy management in a capital breeder. *Journal of Animal Ecology*, *89*(11), 2461–2472. https://doi.org/10.1111/1365-2656.13312

Table S2. All studies used in Figure 1 (n = 103).

Allen, A. S., Read, A. J., Shorter, K. A., Gabaldon, J., Blawas, A. M., Rocho-Levine, J., & Fahlman, A. (2022). Dynamic body acceleration as a proxy to predict the cost of locomotion in bottlenose dolphins. *Journal of Experimental Biology*, *225*(4), jeb243121. <https://doi.org/10.1242/jeb.243121>

Barceló, G., Pauli, J. N., Alldredge, M., & Karasov, W. H. (2023). Assessment of behavioral energetics model on Puma concolor using doubly labeled water. *Canadian Journal of Zoology*, *101*(3), 199–206. <https://doi.org/10.1139/cjz-2022-0029>

Bayer, T.D. (2024). *Navigating the Costs of Movement: Characterizing Energy Expenditure During Long-Distance Foraging in Greater Spear-Nosed Bats* (MSc thesis, Southeastern Louisiana University).

Becciu, P., Rotics, S., Horvitz, N., Kaatz, M., Fiedler, W., Zurell, D., Flack, A., Jeltsch, F., Wikelski, M., Nathan, R., & Sapir, N. (2020). Causes and consequences of facultative sea crossing in a soaring migrant. *Functional Ecology*, *34*(4), 840–852. <https://doi.org/10.1111/1365-2435.13539>

Bennison, A., Giménez, J., Quinn, J. L., Green, J. A., & Jessopp, M. (2022). A bioenergetics approach to understanding sex differences in the foraging behaviour of a sexually monomorphic species. *Royal Society Open Science*, *9*(1), 210520. <https://doi.org/10.1098/rsos.210520>

Benoit, L., Hewison, A. J. M., Coulon, A., Debeffe, L., Grémillet, D., Ducros, D., Cargnelutti, B., Chaval, Y., & Morellet, N. (2020). Accelerating across the landscape: The energetic costs of natal dispersal in a large herbivore. *Journal of Animal Ecology*, *89*(1), 173–185. <https://doi.org/10.1111/1365-2656.13098>

Bishop, C.M., Spivey, R.J., Hawkes, L.A., Batbayar, N., Chua, B., Frappell, P.B., Milsom, W.K., Natsagdorj, T., Newman, S.H., Scott, G.R., & Takekawa, J.Y. (2015). The roller coaster flight strategy of bar-headed geese conserves energy during Himalayan migrations. *Science*, **347**, 250-254.

Brown, J.M., Bouten, W., Camphuysen, K.C., Nolet, B.A., & Shamoun‐Baranes, J. (2022). Acceleration as a proxy for energy expenditure in a facultative‐soaring bird: comparing dynamic body acceleration and time‐energy budgets to heart rate. *Functional Ecology*, **36**, 1627-1638.

Brown, J. M., Bouten, W., Camphuysen, K. C. J., Nolet, B. A., & Shamoun-Baranes, J. (2023). Energetic and behavioral consequences of migration: An empirical evaluation in the context of the full annual cycle. *Scientific Reports*, *13*(1), 1210. <https://doi.org/10.1038/s41598-023-28198-8>

Brownscombe, J. W., Cooke, S. J., & Danylchuk, A. J. (2017). Spatiotemporal drivers of energy expenditure in a coastal marine fish. *Oecologia*, *183*(3), 689–699. <https://doi.org/10.1007/s00442-016-3800-5>

Bryce, C. M., Dunford, C. E., Pagano, A. M., Wang, Y., Borg, B. L., Arthur, S. M., & Williams, T. M. (2022). Environmental correlates of activity and energetics in a wide-ranging social carnivore. *Animal Biotelemetry*, *10*(1), 1. <https://doi.org/10.1186/s40317-021-00272-w>

Byrnes, E. E., Hounslow, J. L., Heim, V., White, C. E., Smukall, M. J., Beatty, S. J., & Gleiss, A. C. (2025). Intraspecific scaling of home range size and its bioenergetic association. *Ecology*, *106*(2), e70003. <https://doi.org/10.1002/ecy.70003>

Cade, D. E., Levenson, J. J., Cooper, R., de la Parra, R., Webb, D. H., & Dove, A. D. M. (2020). Whale sharks increase swimming effort while filter feeding, but appear to maintain high foraging efficiencies. *Journal of Experimental Biology*, *223*(11), jeb224402. <https://doi.org/10.1242/jeb.224402>

Cecere, J. G., De Pascalis, F., Imperio, S., Ménard, D., Catoni, C., Griggio, M., & Rubolini, D. (2020). Inter-individual differences in foraging tactics of a colonial raptor: Consistency, weather effects, and fitness correlates. *Movement Ecology*, *8*(1), 28. <https://doi.org/10.1186/s40462-020-00206-w>

Chakravarty, P., Cozzi, G., Scantlebury, D. M., Ozgul, A., & Aminian, K. (2023). Combining accelerometry with allometry for estimating daily energy expenditure in joules when in-lab calibration is unavailable. *Movement Ecology*, *11*(1), 29. <https://doi.org/10.1186/s40462-023-00395-0>

Chimienti, M., Desforges, J.-P., Beumer, L. T., Nabe-Nielsen, J., van Beest, F. M., & Schmidt, N. M. (2020). Energetics as common currency for integrating high resolution activity patterns into dynamic energy budget-individual based models. *Ecological Modelling*, *434*, 109250. <https://doi.org/10.1016/j.ecolmodel.2020.109250>

Collins, P. M., Halsey, L. G., Arnould, J. P. Y., Shaw, P. J. A., Dodd, S., & Green, J. A. (2016). Energetic consequences of time-activity budgets for a breeding seabird. *Journal of Zoology*, *300*(3), 153–162. <https://doi.org/10.1111/jzo.12370>

Conners, M.G., Green, J.A., Phillips, R.A., Orben, R.A., Cui, C., Djurić, P.M., Heywood, E., Vyssotski, A.L., & Thorne, L.H. (2024). Dynamic soaring decouples dynamic body acceleration and energetics in albatrosses. *Journal of Experimental Biology*, **227**, jeb247431.

Cowan, M. A., Dunlop, J. A., Gibson, L. A., Moore, H. A., Setterfield, S. A., & Nimmo, D. G. (2024). Movement ecology of an endangered mesopredator in a mining landscape. *Movement Ecology*, *12*(1), 5. <https://doi.org/10.1186/s40462-023-00439-5>

Creel, S., Redcliffe, J., Goodheart, B., Reyes de Merkle, J., Mwape, H., Matsushima, S., Dart, C., Banda, K., Mayani, B., Njobvu, J., Kabungo, R., Mungolo, M., Kabwe, R., Kaseketi, E., Donald, W., Kaluka, A., Chifunte, C., Maimbo, H., Plankenhorn, L., … Wilson, R. P. (2025). Prey depletion, interspecific competition, and the energetics of hunting in endangered African wild dogs, Lycaon pictus. *Proceedings of the National Academy of Sciences*, *122*(6), e2414772122. <https://doi.org/10.1073/pnas.2414772122>

Cunningham, J. T., Le Vaillant, M., Gaston, A. J., Ropert-Coudert, Y., Kato, A., Jacobs, S. R., & Elliott, K. H. (2017). Reduced activity in middle-aged thick-billed murres: Evidence for age related trends in fine-scale foraging behaviour. *Animal Behaviour*, *126*, 271–280. <https://doi.org/10.1016/j.anbehav.2017.02.010>

Cunningham, S. A., Schafer, T. L. J., Wikle, C. K., VonBank, J. A., Ballard, B. M., Cao, L., Bearhop, S., Fox, A. D., Hilton, G. M., Walsh, A. J., Griffin, L. R., & Weegman, M. D. (2023). Time-varying effects of local weather on behavior and probability of breeding deferral in two Arctic-nesting goose populations. *Oecologia*, *201*(2), 369–383. <https://doi.org/10.1007/s00442-022-05300-x>

Dalton, A. J. M., Rosen, D. A. S., & Trites, A. W. (2014). Season and time of day affect the ability of accelerometry and the doubly labeled water methods to measure energy expenditure in northern fur seals (*Callorhinus ursinus*). *Journal of Experimental Marine Biology and Ecology*, *452*, 125–136. <https://doi.org/10.1016/j.jembe.2013.12.014>

Del Caño, M., Quintana, F., Yoda, K., Dell’Omo, G., Blanco, G. S., & Gómez-Laich, A. (2021). Fine-scale body and head movements allow to determine prey capture events in the Magellanic Penguin (Spheniscus magellanicus). *Marine Biology*, *168*(6), 84. <https://doi.org/10.1007/s00227-021-03892-1>

Dickinson, E. R., Stephens, P. A., Marks, N. J., Wilson, R. P., & Scantlebury, D. M. (2021). Behaviour, temperature and terrain slope impact estimates of energy expenditure using oxygen and dynamic body acceleration. *Animal Biotelemetry*, *9*(1), 47. <https://doi.org/10.1186/s40317-021-00269-5>

Dunford, C. E., Marks, N. J., Wilmers, C. C., Bryce, C. M., Nickel, B., Wolfe, L. L., Scantlebury, D. M., & Williams, T. M. (2020). Surviving in steep terrain: A lab-to-field assessment of locomotor costs for wild mountain lions (Puma concolor). *Movement Ecology*, *8*(1), 34. <https://doi.org/10.1186/s40462-020-00215-9>

Dupuis, B., Kato, A., Hicks, O., Wisniewska, D. M., Marciau, C., Angelier, F., Ropert-Coudert, Y., & Chimienti, M. (2024). Innovative use of depth data to estimate energy intake and expenditure in Adélie penguins. *Journal of Experimental Biology*, *227*(23), jeb249201. <https://doi.org/10.1242/jeb.249201>

Duriez, O., Kato, A., Tromp, C., Dell'Omo, G., Vyssotski, A.L., Sarrazin, F., & Ropert-Coudert, Y., (2014). How cheap is soaring flight in raptors? A preliminary investigation in freely-flying vultures. *PloS one*, **9**, e84887.

Elliott, K.H., Le Vaillant, M., Kato, A., Speakman, J.R., & Ropert-Coudert, Y. (2013). Accelerometry predicts daily energy expenditure in a bird with high activity levels. *Biology letters*, **9**(1), 20120919.

Enstipp, M. R., Ballorain, K., Ciccione, S., Narazaki, T., Sato, K., & Georges, J.-Y. (2016). Energy expenditure of adult green turtles (Chelonia mydas) at their foraging grounds and during simulated oceanic migration. *Functional Ecology*, *30*(11), 1810–1825. <https://doi.org/10.1111/1365-2435.12667>

Fahlman, A., Wilson, R., Svärd, C., Rosen, D., & Trites, A. (2008). Activity and diving metabolism correlate in Steller sea lion Eumetopias jubatus. *Aquatic Biology*, *2*, 75–84. <https://doi.org/10.3354/ab00039>

Finnegan, S. P., Pagano, A. M., Svoboda, N. J., Schooler, S. L., & Belant, J. L. (2023). Energy landscapes of Kodiak brown bears: A comparison of accelerometer and global positioning system-derived estimates. *Animal Biotelemetry*, *11*(1), 7. <https://doi.org/10.1186/s40317-023-00319-0>

Flack, A., Fiedler, W., Blas, J., Pokrovsky, I., Kaatz, M., Mitropolsky, M., Aghababyan, K., Fakriadis, I., Makrigianni, E., Jerzak, L., Azafzaf, H., Feltrup-Azafzaf, C., Rotics, S., Mokotjomela, T. M., Nathan, R., & Wikelski, M. (2016). Costs of migratory decisions: A comparison across eight white stork populations. *Science Advances*, *2*(1), e1500931. <https://doi.org/10.1126/sciadv.1500931>

Flack, A., Schaeffer, P.J., Taylor, J.R., Müller, I., Wikelski, M., & Fiedler, W. (2020). Daily energy expenditure in white storks is lower after fledging than in the nest. *Journal of Experimental Biology*, **223**, jeb219337.

Gabaldon, J. T., Zhang, D., Rocho-Levine, J., Moore, M. J., van der Hoop, J., Barton, K., & Shorter, K. A. (2022). Tag-based estimates of bottlenose dolphin swimming behavior and energetics. *Journal of Experimental Biology*, *225*(22), jeb244599. <https://doi.org/10.1242/jeb.244599>

Gatt, M. C., Quetting, M., Cheng, Y., & Wikelski, M. (2020). Dynamic body acceleration increases by 20% during flight ontogeny of greylag geese Anser anser. *Journal of Avian Biology*, *51*(2). <https://doi.org/10.1111/jav.02235>

Gómez Laich, A., Wilson, R. P., Gleiss, A. C., Shepard, E. L. C., & Quintana, F. (2011). Use of overall dynamic body acceleration for estimating energy expenditure in cormorants: Does locomotion in different media affect relationships? *Journal of Experimental Marine Biology and Ecology*, *399*(2), 151–155. <https://doi.org/10.1016/j.jembe.2011.01.008>

Gómez-Laich, A., Wilson, R. P., Shepard, E. L. C., & Quintana, F. (2013). Energy expenditure and food consumption of foraging Imperial cormorants in Patagonia, Argentina. *Marine Biology*, *160*(7), 1697–1707. <https://doi.org/10.1007/s00227-013-2222-8>

Gonçalves, P., Magalhães, J., & Corujo, D. (2024). Estimating the Energy Expenditure of Grazing Farm Animals Based on Dynamic Body Acceleration. *Animals*, *14*(15), Article 15. <https://doi.org/10.3390/ani14152140>

Gou, X., Rong, Y., Cheng, Y., Li, P., Zhang, H., Zhang, Z., Huo, T., Li, G., & Liang, J. (n.d.). *Greater impact of energy expenditure over harvest rate on resource conversion efficiency as herbivores grow*. Retrieved March 6, 2025, from <https://www.authorea.com/doi/full/10.22541/au.171355880.00343429?commit=360d11480636bc8736a635854a28cd78a96a3755>

Grémillet, D., Lescroël, A., Ballard, G., Dugger, K. M., Massaro, M., Porzig, E. L., & Ainley, D. G. (2018). Energetic fitness: Field metabolic rates assessed via 3D accelerometry complement conventional fitness metrics. *Functional Ecology*, *32*(5), 1203–1213. <https://doi.org/10.1111/1365-2435.13074>

Guillemette, M. and Butler, P.J., 2012. Seasonal variation in energy expenditure is not related to activity level or water temperature in a large diving bird. *Journal of Experimental Biology*, *215*(18), pp.3161-3168.

Gutzler, B. C., & Watson III, W. H. (2022). Measurements of American lobster heart rate and movements in the wild using a low-cost open source datalogger. *Marine Biology*, *169*(4), 52. https://doi.org/10.1007/s00227-022-04046-7Barracho, T., Hatch, S.A., Kotzerka, J., Garthe, S., Schraft, H.A., Whelan, S. and Elliott, K.H., 2024. Survival costs of reproduction are independent of energy costs in a seabird, the pelagic cormorant. *Ecology and Evolution*, *14*(7), p.e11414.

Hicks, O., Burthe, S., Daunt, F., Butler, A., Bishop, C., & Green, J.A. (2017). Validating accelerometry estimates of energy expenditure across behaviours using heart rate data in a free-living seabird. *Journal of Experimental Biology*, **220**(10), 1875-1881.

Hicks, O., Kato, A., Angelier, F., Wisniewska, D.M., Hambly, C., Speakman, J.R., Marciau, C., & Ropert-Coudert, Y. (2020). Acceleration predicts energy expenditure in a fat, flightless, diving bird. *Scientific Reports*, **10**, 21493.

Hubel, T. Y., Myatt, J. P., Jordan, N. R., Dewhirst, O. P., McNutt, J. W., & Wilson, A. M. (2016). Energy cost and return for hunting in African wild dogs and cheetahs. *Nature Communications*, *7*(1), 11034. <https://doi.org/10.1038/ncomms11034>

Hurme, E., Lenzi, I., Wikelski, M., Wild, T. A., & Dechmann, D. K. N. (2025). Bats surf storm fronts during spring migration. *Science*, *387*(6729), 97–102. <https://doi.org/10.1126/science.ade7441>

Jeanniard‐du‐Dot, T., Guinet, C., Arnould, J.P., Speakman, J.R., & Trites, A.W. (2017). Accelerometers can measure total and activity‐specific energy expenditures in free‐ranging marine mammals only if linked to time‐activity budgets. *Functional Ecology*, **31**, 377-386.

Kirchner, T. (2024). Do you mind? Using biologging tools to study anthropogenic disturbance effects on wildlife behavior and energetics–a case study on moose. Ph.D. thesis, Inland Norway University of Applied Sciences.

Kristiansen, M. (2014). Can energy expenditure of free-ranging kittiwakes be estimated by body acceleration? Master's thesis, University of the Arctic.

Leimgruber, P., Songsasen, N., Stabach, J.A., Horning, M., Reed, D., Buk, T., Harwood, A., Layman, L., Mathews, C., Vance, M., & Marinari, P. (2023). Providing baseline data for conservation–Heart rate monitoring in captive scimitar-horned oryx. *Frontiers in Physiology*, **14**, 1079008.

Ladds, M. A., Salton, M., Hocking, D. P., McIntosh, R. R., Thompson, A. P., Slip, D. J., & Harcourt, R. G. (2018). Using accelerometers to develop time-energy budgets of wild fur seals from captive surrogates. *PeerJ*, *6*, e5814. <https://doi.org/10.7717/peerj.5814>

Masello, J. F., Barbosa, A., Kato, A., Mattern, T., Medeiros, R., Stockdale, J. E., Kümmel, M. N., Bustamante, P., Belliure, J., Benzal, J., Colominas-Ciuró, R., Menéndez-Blázquez, J., Griep, S., Goesmann, A., Symondson, W. O. C., & Quillfeldt, P. (2021). How animals distribute themselves in space: Energy landscapes of Antarctic avian predators. *Movement Ecology*, *9*(1), 24. <https://doi.org/10.1186/s40462-021-00255-9>

Menzies, A. (2021). Warm bodies in cold places: thermoregulation, activity, and energy expenditure of boreal homeotherms in winter. PhD thesis. McGill University.

Menzies, A. K., Studd, E. K., Seguin, J. L., Derbyshire, R. E., Murray, D. L., Boutin, S., & Humphries, M. M. (2022). Activity, heart rate, and energy expenditure of a cold-climate mesocarnivore, the Canada lynx (Lynx canadensis). *Canadian Journal of Zoology*, *100*(4), 261–272. <https://doi.org/10.1139/cjz-2021-0142>

Miwa, M., Oishi, K., Anzai, H., Kumagai, H., Ieiri, S., & Hirooka, H. (2017). Estimation of the energy expenditure of grazing ruminants by incorporating dynamic body acceleration into a conventional energy requirement system 1. *Journal of Animal Science*, *95*(2), 901–909. <https://doi.org/10.2527/jas2016.0749>

Mizrahy-Rewald, O., Perinot, E., Fritz, J., Vyssotski, A.L., Fusani, L., Voelkl, B., & Ruf, T., (2022). Empirical evidence for energy efficiency using intermittent gliding flight in northern bald ibises. *Frontiers in Ecology and Evolution*, **10**, 891079.

Moore, H. A., Diete, R. L., Indigo, N. L., Cowan, M. A., Trewella, G. J., & Nimmo, D. G. (2024). Midnight siesta: Bimodal temporal activity observed in an endangered marsupial predator. *Austral Ecology*, *49*(4), e13521. <https://doi.org/10.1111/aec.13521>

Morant, J., Scacco, M., Safi, K., Gómez, J. M. A., Álvarez, T., Sánchez, Á., Phipps, W. L., Alanís, I. C., García, J., Prieta, J., Zuberogoitia, I., & López-López, P. (2022). Environmental and social correlates, and energetic consequences of fitness maximisation on different migratory behaviours in a long-lived scavenger. *Behavioral Ecology and Sociobiology*, *76*(8), 111. <https://doi.org/10.1007/s00265-022-03223-4>

Morgan, A., Christensen, C., Bracken, A. M., O’Riain, M. J., King, A. J., & Fürtbauer, I. (2023). Effects of accelerometry-derived physical activity energy expenditure on urinary C-peptide levels in a wild primate (*Papio ursinus*). *Hormones and Behavior*, *152*, 105355. <https://doi.org/10.1016/j.yhbeh.2023.105355>

Nathan, R., Spiegel, O., Fortmann-Roe, S., Harel, R., Wikelski, M., & Getz, W. M. (2012). Using tri-axial acceleration data to identify behavioral modes of free-ranging animals: General concepts and tools illustrated for griffon vultures. *Journal of Experimental Biology*, *215*(6), 986–996. <https://doi.org/10.1242/jeb.058602>

Niccolai, L.J., Devineau, O., Thiel, A., Zimmermann, B., & Evans, L.A. (2024). Connecting the dots: relationship between heart rate and overall dynamic body acceleration in free-ranging cattle. *Conservation Physiology*, **12**, coae085.

Nickel, B. A., Suraci, J. P., Nisi, A. C., & Wilmers, C. C. (2021). Energetics and fear of humans constrain the spatial ecology of pumas. *Proceedings of the National Academy of Sciences*, *118*(5), e2004592118. <https://doi.org/10.1073/pnas.2004592118>

Nielsen, L. R., Tervo, O. M., Blackwell, S. B., Heide-Jørgensen, M. P., & Ditlevsen, S. (2023). Using quantile regression and relative entropy to assess the period of anomalous behavior of marine mammals following tagging. *Ecology and Evolution*, *13*(4), e9967. <https://doi.org/10.1002/ece3.9967>

Olejarz, A., Faltusová, M., Börger, L., Güldenpfennig, J., Jarský, V., Ježek, M., Mortlock, E., Silovský, V., & Podgórski, T. (2023). Worse sleep and increased energy expenditure yet no movement changes in sub-urban wild boar experiencing an influx of human visitors (anthropulse) during the COVID-19 pandemic. *Science of The Total Environment*, *879*, 163106. <https://doi.org/10.1016/j.scitotenv.2023.163106>

O’Mara, M. T., Scharf, A. K., Fahr, J., Abedi-Lartey, M., Wikelski, M., Dechmann, D. K. N., & Safi, K. (2019). Overall Dynamic Body Acceleration in Straw-Colored Fruit Bats Increases in Headwinds but Not With Airspeed. *Frontiers in Ecology and Evolution*, *7*. <https://doi.org/10.3389/fevo.2019.00200>

Pagano, A.M., & Williams, T.M. (2019). Estimating the energy expenditure of free‐ranging polar bears using tri‐axial accelerometers: A validation with doubly labeled water. *Ecology and Evolution*, **9**, 4210-4219.

Papastamatiou, Y. P., Iosilevskii, G., Leos-Barajas, V., Brooks, E. J., Howey, L. A., Chapman, D. D., & Watanabe, Y. Y. (2018). Optimal swimming strategies and behavioral plasticity of oceanic whitetip sharks. *Scientific Reports*, *8*(1), 551. <https://doi.org/10.1038/s41598-017-18608-z>

Payne, N. L., Gillanders, B. M., Seymour, R. S., Webber, D. M., Snelling, E. P., & Semmens, J. M. (2011). Accelerometry estimates field metabolic rate in giant Australian cuttlefish Sepia apama during breeding. *Journal of Animal Ecology*, *80*(2), 422–430. <https://doi.org/10.1111/j.1365-2656.2010.01758.x>

Perinot, E., Rewald, O. M., Fritz, J., Nobile, M. S., Vyssotski, A., Ruf, T., Fusani, L., & Voelkl, B. (2024). Small energy benefits of in-wake flying in long-duration migratory flights. *Proceedings of the Royal Society B: Biological Sciences*, *291*(2030), 20241173. <https://doi.org/10.1098/rspb.2024.1173>

Rezende, G.C., Cruz-Neto, A.P., Börger, L., Redcliffe, J., Hambly, C., Speakman, J.R., Garbino, G.S., Pissinatti, A., Moreira, S.B., Wilson, R., & Culot, L. (2023). Validating Dynamic Body Acceleration metrics as a measure of energy expenditure in a Neotropical primate. *bioRxiv*, 2023-06.

Rotics, S., Kaatz, M., Resheff, Y. S., Turjeman, S. F., Zurell, D., Sapir, N., Eggers, U., Flack, A., Fiedler, W., Jeltsch, F., Wikelski, M., & Nathan, R. (2016). The challenges of the first migration: Movement and behaviour of juvenile vs. adult white storks with insights regarding juvenile mortality. *Journal of Animal Ecology*, *85*(4), 938–947. <https://doi.org/10.1111/1365-2656.12525>

Scacco, M., Flack, A., Duriez, O., Wikelski, M., & Safi, K. (2019). Static landscape features predict uplift locations for soaring birds across Europe. *Royal Society Open Science*, *6*(1), 181440. <https://doi.org/10.1098/rsos.181440>

Schindler, A. R., Fox, A. D., Wikle, C. K., Ballard, B. M., Walsh, A. J., Kelly, S. B. A., Cao, L., Griffin, L. R., & Weegman, M. D. (2024). Energetic trade-offs in migration decision-making, reproductive effort and subsequent parental care in a long-distance migratory bird. *Proceedings of the Royal Society B: Biological Sciences*, *291*(2017), 20232016. <https://doi.org/10.1098/rspb.2023.2016>

Shepard, E. L. C., Wilson, R. P., Quintana, F., Gómez Laich, A., & Forman, D. W. (2009). Pushed for time or saving on fuel: Fine-scale energy budgets shed light on currencies in a diving bird. *Proceedings of the Royal Society B: Biological Sciences*, *276*(1670), 3149–3155. <https://doi.org/10.1098/rspb.2009.0683>

Shuert, C. R., Marcoux, M., Hussey, N. E., Watt, C. A., & Auger-Méthé, M. (n.d.). *Assessing the post-release effects of capture, handling and placement of satellite telemetry devices on narwhal (Monodon monoceros) movement behaviour*. Retrieved March 11, 2025, from <https://dx.doi.org/10.1093/conphys/coaa128>

Skinner, J. P., Mitani, Y., Burkanov, V. N., & Andrews, R. D. (2014). Proxies of food intake and energy expenditure for estimating the time–energy budgets of lactating northern fur seals *Callorhinus ursinus*. *Journal of Experimental Marine Biology and Ecology*, *461*, 107–115. <https://doi.org/10.1016/j.jembe.2014.08.002>

Sotillo, A., Baert, J. M., Müller, W., Stienen, E. W. M., Soares, A. M. V. M., & Lens, L. (2019). Time and energy costs of different foraging choices in an avian generalist species. *Movement Ecology*, *7*(1), 41. <https://doi.org/10.1186/s40462-019-0188-y>

Ste-Marie, E., Grémillet, D., Fort, J., Patterson, A., Brisson-Curadeau, É., Clairbaux, M., Perret, S., Speakman, J. R., & Elliott, K. H. (2022). Accelerating animal energetics: High dive costs in a small seabird disrupt the dynamic body acceleration–energy expenditure relationship. *Journal of Experimental Biology*, *225*(12), jeb243252. <https://doi.org/10.1242/jeb.243252>

Ste-Marie, E., Watanabe, Y. Y., Semmens, J. M., Marcoux, M., & Hussey, N. E. (2022). Life in the slow lane: Field metabolic rate and prey consumption rate of the Greenland shark (Somniosus microcephalus) modelled using archival biologgers. *Journal of Experimental Biology*, *225*(7), jeb242994. <https://doi.org/10.1242/jeb.242994>

Stothart, M.R., Elliott, K.H., Wood, T., Hatch, S.A., & Speakman, J.R. (2016). Counting calories in cormorants: dynamic body acceleration predicts daily energy expenditure measured in pelagic cormorants. *Journal of Experimental Biology*, **219**(14), 2192-2200.

Sutton, G.J., Angel, L.P., Speakman, J.R., & Arnould, J.P. (2023). Determining energy expenditure in a large seabird using accelerometry. *Journal of Experimental Biology*, **226**(23).

Sutton, G.J., Botha, J.A., Speakman, J.R., & Arnould, J.P.Y. (2021). Validating accelerometry-derived proxies of energy expenditure using the doubly labelled water method in the smallest penguin species. *Biology Open*, **10**(4), bio055475.

Tatler, J., Currie, S. E., Cassey, P., Scharf, A. K., Roshier, D. A., & Prowse, T. A. A. (2021). Accelerometer informed time-energy budgets reveal the importance of temperature to the activity of a wild, arid zone canid. *Movement Ecology*, *9*(1), 11. <https://doi.org/10.1186/s40462-021-00246-w>

Tremblay, F., Choy, E.S., Whelan, S., Hatch, S., & Elliott, K.H. (2024). Time-energy budgets outperform dynamic body acceleration in predicting daily energy expenditure in kittiwakes, and estimate a very low cost of gliding flight relative to flapping flight. *Journal of Experimental Biology*, **227**, jeb247176.

Trondrud, L.M., Pigeon, G., Król, E., Albon, S., Evans, A.L., Arnold, J.W., Hambly, C., Irvine, R.J., Ropstad, E., Stien, A., & Veiberg, V., 2021. Fat storage influences fasting endurance more than body size in an ungulate. *Functional Ecology*, **35**, 1470-1480.

Trondrud, L.M., Pigeon, G., Albon, S., Arnold, W., Evans, A.L., Irvine, R.J., Król, E., Ropstad, E., Stien, A., Veiberg, V., & Speakman, J.R. (2021). Determinants of heart rate in Svalbard reindeer reveal mechanisms of seasonal energy management. *Philosophical Transactions of the Royal Society B*, **376**, 20200215.

Udyawer, V., Simpfendorfer, C. A., Heupel, M. R., & Clark, T. D. (2017). Temporal and spatial activity-associated energy partitioning in free-swimming sea snakes. *Functional Ecology*, *31*(9), 1739–1749. <https://doi.org/10.1111/1365-2435.12882>

van Oordt, F., Silva, J., Patterson, A., & Elliott, K.H. (2024). Plunge-diving into dynamic body acceleration and energy expenditure in the Peruvian booby. *Journal of Experimental Biology*, **227**, jeb249555.

Van Walsum, T. A., Perna, A., Bishop, C. M., Murn, C. P., Collins, P. M., Wilson, R. P., & Halsey, L. G. (2020). Exploring the relationship between flapping behaviour and accelerometer signal during ascending flight, and a new approach to calibration. *Ibis*, *162*(1), 13–26. <https://doi.org/10.1111/ibi.12710>

Waller, M. J., Queiroz, N., da Costa, I., Cidade, T., Loureiro, B., Womersley, F. C., Fontes, J., Afonso, P., Macena, B. C. L., Loveridge, A., Humphries, N. E., Southall, E. J., & Sims, D. W. (2023). Direct measurement of cruising and burst swimming speeds of the shortfin mako shark (Isurus oxyrinchus) with estimates of field metabolic rate. *Journal of Fish Biology*, *103*(5), 864–883. <https://doi.org/10.1111/jfb.15475>

Wang, Y., Smith, J. A., & Wilmers, C. C. (2017). Residential development alters behavior, movement, and energetics in an apex predator, the puma. *PLOS ONE*, *12*(10), e0184687. <https://doi.org/10.1371/journal.pone.0184687>

Watanabe, Y. Y., Payne, N. L., Semmens, J. M., Fox, A., & Huveneers, C. (2019). Swimming strategies and energetics of endothermic white sharks during foraging. *Journal of Experimental Biology*, *222*(4), jeb185603. <https://doi.org/10.1242/jeb.185603>

Weimerskirch, H., Bishop, C., Jeanniard-du-Dot, T., Prudor, A., & Sachs, G. (2016). Frigate birds track atmospheric conditions over months-long transoceanic flights. *Science*, **353**, 74-78.

Williams, T.M., Fuiman, L.A., Horning, M., & Davis, R.W. (2004). The cost of foraging by a marine predator, the Weddell seal *Leptonychotes weddellii*: pricing by the stroke. *Journal of experimental biology*, **207**(6), 973-982.

Williams, T. M., Peter-Heide Jørgensen, M., Pagano, A. M., & Bryce, C. M. (2020). Hunters versus hunted: New perspectives on the energetic costs of survival at the top of the food chain. *Functional Ecology*, *34*(10), 2015–2029. <https://doi.org/10.1111/1365-2435.13649>

Williams, T. M., Wolfe, L., Davis, T., Kendall, T., Richter, B., Wang, Y., Bryce, C., Elkaim, G. H., & Wilmers, C. C. (2014). Instantaneous energetics of puma kills reveal advantage of felid sneak attacks. *Science*, *346*(6205), 81–85. <https://doi.org/10.1126/science.1254885>

Wilmers, C. C., Isbell, L. A., Suraci, J. P., & Williams, T. M. (2017). Energetics-informed behavioral states reveal the drive to kill in African leopards. *Ecosphere*, *8*(6), e01850. <https://doi.org/10.1002/ecs2.1850>

Wilson, R. P., Reynolds, S. D., Potts, J. R., Redcliffe, J., Holton, M., Buxton, A., Rose, K., & Norman, B. M. (2022). Highlighting when animals expend excessive energy for travel using dynamic body acceleration. *iScience*, *25*(9). <https://doi.org/10.1016/j.isci.2022.105008>

Wilson, R. P., White, C. R., Quintana, F., Halsey, L. G., Liebsch, N., Martin, G. R., & Butler, P. J. (2006). Moving towards acceleration for estimates of activity-specific metabolic rate in free-living animals: The case of the cormorant. *Journal of Animal Ecology*, *75*(5), 1081–1090. <https://doi.org/10.1111/j.1365-2656.2006.01127.x>

Wilson, R., Shepard, E., Gómez Laich, A., Frere, E., & Quintana, F. (2010). Pedalling downhill and freewheeling up; a penguin perspective on foraging. *Aquatic Biology*, *8*, 193–202. <https://doi.org/10.3354/ab00230>

Wilson, S. M., Hinch, S. G., Eliason, E. J., Farrell, A. P., & Cooke, S. J. (2013). Calibrating acoustic acceleration transmitters for estimating energy use by wild adult Pacific salmon. *Comparative Biochemistry and Physiology Part A: Molecular & Integrative Physiology*, *164*(3), 491–498. <https://doi.org/10.1016/j.cbpa.2012.12.002>
